# A topical review of the feasibility and reliability of ambulance-based telestroke

**DOI:** 10.3389/fstro.2024.1363140

**Published:** 2024-03-28

**Authors:** Sherita N. Chapman, Theandra Madu, Nisha Dabhi, Jackson A. Narrett, Necrisha N. Roach, Haydon M. Pitchford, Marcus C. Divers, Andrew M. Southerland

**Affiliations:** ^1^Department of Neurology, University of Virginia, Charlottesville, VA, United States; ^2^Department of Neurology, Central Virginia VA Healthcare System, Richmond, VA, United States; ^3^Department of Neurology, Perelman School of Medicine, University of Pennsylvania, Philadelphia, PA, United States; ^4^Department of Neurology, Yale University School of Medicine, New Haven, CT, United States; ^5^All-In-Solutions, LLC, Fredericksburg, VA, United States; ^6^Virginia Health Information, Richmond, VA, United States; ^7^Department of Public Health Sciences, University of Virginia, Charlottesville, VA, United States

**Keywords:** stroke, telemedicine, telestroke, mobile health, emergency medical services, prehospital

## Abstract

**Background:**

Ambulance-based telemedicine is an innovative strategy through which transport time can be used to rapidly and accurately triage stroke patients (i.e., mobile telestroke). The acute phase of stroke is a time-sensitive emergency, and delays in care during this phase worsen outcomes. In this literature review, we analyzed studies that investigated the feasibility and reliability of ambulance based telestroke.

**Methods:**

We followed PRISMA guidelines to perform a keyword-based search of PubMed, Web of Science, CINHAL, and Academic Search Complete databases. We reviewed references of search-identified articles to screen for additional articles. Articles for inclusion were selected according to author consensus in consideration of the studies' investigation of feasibility, reliability, or validity of ambulance-based telestroke.

**Results:**

We identified 67 articles for secondary screening from which 19 articles were selected for full text review. The selected studies reported diverse methods of development, implementation, and assessment of ambulance-based telestroke systems. Although the methods and results varied among these studies, most concluded that the implementation of ambulance based telestroke is feasible.

**Conclusion:**

This topical review suggests that ambulance based telestroke is a feasible method for enhanced prehospital stroke care in a variety of settings. Further prospective research is needed to assess the real-world challenges and to identify additional strategies that bolster rapid and accurate prehospital assessment of acute stroke patients.

## Introduction

Stroke is a leading cause of death and disability worldwide (Feigin et al., 2017; GBD 2017 Causes of Death Collaborators, 2018; Tsao et al., 2023). The most effective treatment for acute ischemic stroke is early reperfusion therapy, for which earlier treatment is associated with better recovery outcomes (Saver et al., 2016; Kim et al., 2017; Agarwal et al., 2018). Pre- and in-hospital factors including limited resources, pre-hospital diagnostic inaccuracy, geographic barriers, and lack of expertise can delay treatment and contribute to poor outcomes (National Institute of Neurological Disorders and Stroke, 2022; Chari et al., 2023; Oostema et al., 2023; Zachrison et al., 2023). The application of telemedicine to stroke care brings vascular neurology expertise to the emergency room setting (i.e., telestroke). Hospitals that access stroke expertise via telestroke have increased administration of thrombolysis to eligible patients and decreased door to treatment times, and randomized trials have shown that video-based telemedicine consultation outperformed telephone-only consultation in terms of accurate thrombolysis decision making (Meyer et al., 2008; Demaerschalk et al., 2012; Bladin and Cadilhac, 2014; Barlinn et al., 2017).

Approximately half of all patients with acute stroke present to the healthcare system via emergency medical services (EMS; Adeoye et al., 2009). Therefore, stroke recognition by EMS providers in the field is a critical early step in the stroke care continuum. Despite the development of multiple validated prehospital stroke scales, the accuracy of stroke assessment by EMS providers varies widely (Brandler et al., 2014; Pitchford et al., 2019). In addition, advancements in stroke treatment to include endovascular therapy for patients with large vessel occlusion (LVO) have presented an additional need for innovation in prehospital stroke diagnosis and triage (Smith et al., 2018; Keigher, 2020; Jauch et al., 2021). A 2009 scientific statement from the American Heart Association–American Stroke Association suggested that ambulance-based telemedicine for stroke might offer a novel solution to improving the accuracy of prehospital stroke assessment and diagnosis and the timeliness of treatment but that further research was needed (Schwamm et al., 2009). Early research in ambulance-based telestroke yielded conflicting results, for which limited technological and broadband capabilities were implicated as potential barriers (LaMonte et al., 2000, 2004; Liman et al., 2012). However, recent advances in wireless cellular technologies and mobile teleconferencing platforms have increased the capacity for implementing an ambulance-based telestroke system (Akbik et al., 2017). These findings indicate the potential of ambulance-based telestroke as an ultra-early, reliable, and valid prehospital stroke assessment method that can facilitate appropriate triage, decrease time to treatment, and improve patient outcomes.

In this context, we performed a topical literature review using a systematic search strategy to summarize the body of research on the feasibility and reliability of ambulance-based, prehospital telestroke assessment. The goals of this review were to describe previously studied ambulance-based telestroke technologies, assess the accuracy and reliability of ambulance-based telestroke clinical assessments, and identify persistent knowledge gaps and future research opportunities.

## Materials and methods

**Figure 1:**
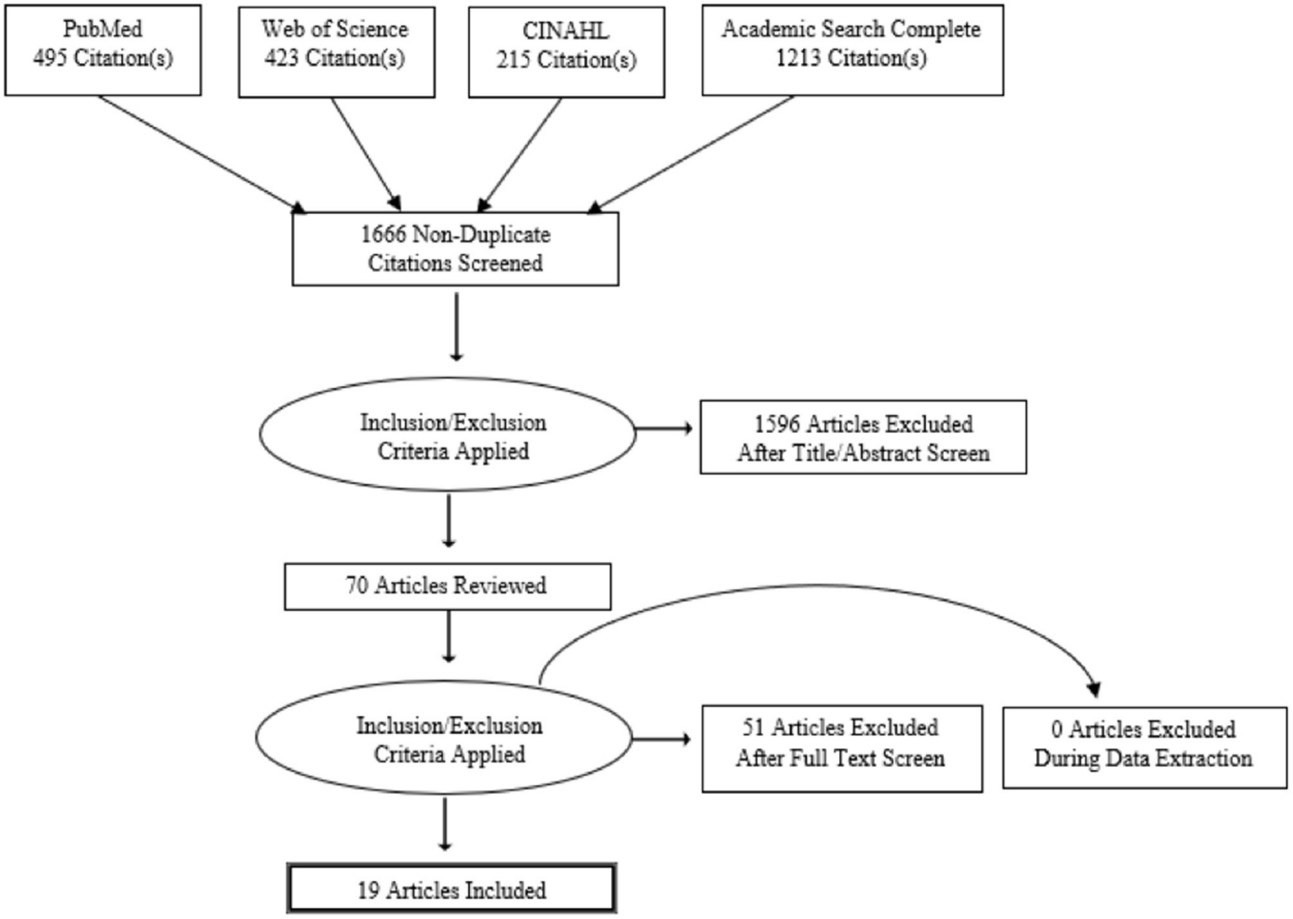
Article review process flow chart.

Using the query string “[telestroke or (telemed^*^ AND stroke) or (telehealth AND stroke)] AND (prehospital OR ambulanc^*^ OR mobile OR EMS OR emergency medical services),” we searched the MEDLINE/PubMed, Web of Science, CINAHL, and Academic Search Complete databases for articles published through February 2022. Our search followed the Preferred Reporting Items of Systematic Review and Meta-Analyses (PRISMA) methodology. Search results were curated to include only publications in English (Figure 1), manually de-duplicated, and uploaded to Rayyan, a web-based collaborative abstract screening platform.

To identify candidate articles eligible for full-text screening, four of the authors of the current study (HMP, JAN, MCP, and TCM) independently applied the predetermined study inclusion and exclusion criteria (Table 1) during screening of the articles' titles and abstracts. Five of the authors (HXP, JAN, MCP, TCM, and SNC) then independently applied the inclusion and exclusion criteria during full-text review of selected articles and evaluated whether the candidate study reported quantified data regarding the feasibility, reliability, or validity of prehospital telestroke technology or assessment. Final decisions regarding an article's inclusion for review were unanimous, and consensus was achieved through collaborative review and discussion.

**Table 1:** Criteria applied to articles identified through keyword searches of databases.

**Inclusion criteria^a^**	**Exclusion criteria^b^**
•Article was written in English. •Published in a peer-reviewed journal. •Investigated the feasibility, reliability and/or validity of ambulance-based telestroke technology and assessment in the prehospital setting. •Included an ambulance-based telestroke platform with a patient interface. •Evaluated the feasibility, reliability, or validity of an ambulance-based telestroke platform. •Conducted in a prehospital setting.	•Article was not written in English. •Not a peer-reviewed journal. •Abstract only, case report, other systemic review, commentary, editorial, or protocol. •Involved helicopter or air ambulance. •Evaluated hospital-based telestroke methods. •Unrelated to stroke care •Exclusively investigated teleradiology

^a^The article had to meet all inclusion criteria to be considered for review.

^b^The article had to meet a single exclusion criterion to be eliminated from review.

Labeling of rural vs. urban vs. suburban was defined by the authors.

### Results

The PRISMA flow diagram (Figure 1) graphically displays the systematic review, selection, and inclusion of the 19 studies discussed in this review. Table 2 summarizes the characteristics of these studies (LaMonte et al., 2000, 2004; Bergrath et al., 2012; Liman et al., 2012; Van Hooff et al., 2013; Wu et al., 2014, 2017; Eadie et al., 2015; Belt et al., 2016; Chapman Smith et al., 2016, 2019; Itrat et al., 2016; Lippman et al., 2016; Valenzuela Espinoza et al., 2016; Barrett et al., 2017; Geisler et al., 2019; Johansson et al., 2019; Winter et al., 2019; Al Kasab et al., 2021). The earliest study identified was published in 2000, while 17 (89%) were published between 2010 and 2021 after the advancement of wireless broadband technology with the introduction of 4G. We identified eight studies (42%) that investigated the feasibility and/or reliability of ambulance-based telestroke techniques using only simulated patient encounters (i.e., the role of the patient was played by a healthy volunteer, medical trainee, or standardized actor). Additionally, of the studies reviewed, eight studies (42%) investigated ambulance based telestroke by evaluating only live patients being transported in ambulances. A combination of simulation and live patient encounters occurred in two studies (11%) and one study (5%) investigated technical feasibility only.

**Table 2:** Study characteristics included in the topical review.

**References**	**Location**	**Setting**	**Type of study**	**Transport setting?**
LaMonte et al. (2000)	Baltimore, Maryland, USA	Urban	Simulation and Prospective	Interfacility
LaMonte et al. (2004)	Baltimore, Maryland, USA	Urban	Simulation	Interfacility
Bergrath et al. (2012)	Aachen, Germany	Urban	Prospective	Prehospital
Liman et al. (2012)	Berlin, Germany	Suburban and Urban	Simulation	Prehospital
Van Hooff et al. (2013)	Brussels, Belgium	Suburban and Urban	Simulation	Prehospital
Eadie et al. (2015)	Inverness, United Kingdom	Rural	Simulation	Prehospital
Wu et al. (2014)	Houston, Texas, USA	Suburban and Urban	Simulation	Prehospital
Belt et al. (2016)	Summit, New Jersey, USA	Urban	Prospective	Prehospital
Chapman Smith et al. (2016)	Charlottesville, Virginia and Berkeley, California, USA	Rural and Urban	Simulation	Prehospital
Valenzuela Espinoza et al. (2016)	Brussels, Belgium	Urban	Prospective	Prehospital
Itrat et al. (2016)	Cleveland, Ohio, USA	Rural and Urban	Prospective	Mobile Stroke Unit
Lippman et al. (2016)	Charlottesville, Virginia, USA	Rural	Technical Observation	Prehospital
Barrett et al. (2017)	Jacksonville, Florida, USA	Urban	Simulation and Prospective	Interfacility
Wu et al. (2017)	Houston, Texas USA	Urban	Prospective	Mobile Stroke Unit
Chapman Smith et al. (2019)	Richmond, Virginia, USA	Urban	Simulation	Prehospital
Geisler et al. (2019)	Berlin, Germany	Urban	Prospective	Mobile Stroke Unit
Johansson et al. (2019)	Lund-Malmö, Sweden	Suburban and Urban	Prospective	Prehospital
Winter et al. (2019)	Berlin, Germany	Urban	Simulation	Mobile Stroke Unit
Al Kasab et al. (2021)	Charleston, South Carolina, USA	Rural	Prospective	Prehospital

The geographical settings of each of the studies are demonstrated in Table 2 with over half conducted in North America and in urban settings. While there was no variability in the mode of transport used to bring the patients to the hospital, there were differences in the setting from which patients were selected to participate.

#### Technology

A variety of mobile telestroke hardware, software, and networks were used and are shown in Table 3. These systems included custom telemedicine systems engineered from commercially available devices, mobile telemedicine specific hardware (prototypical or commercially available), and off –the- shelf consumer technology. A vast majority (89%) used synchronous real-time video-conferencing technology. Over half (58%) used a 4G/4G LTE wireless data network. Additional studies used a combination of networks (21%). Six studies reported the price of their telemedicine hardware with a wide range of $565–$72,000 per ambulance. Irrespective of the equipment used, each of the studies experienced some technical difficulties ranging from issues related to transmission instability, deficiencies in audio-video quality, and human error.

**Table 3:** Technology used for ambulance-based telestroke in reviewed studies.

**References**	**System**	**Data transmission method**	**Data network**	**Technical issues reported**
LaMonte et al. (2000)	Existing open system, commercially available components with parallel array of four digital cellular phones	Store and forward	2G	The stability of transmission link remains an issue due to: (1) All connected phones can disconnect simultaneously when traveling through gaps in cellular network coverage. (2) Connected phones can disconnect when traveling from one network cell into another. (3) Reconnection occurs only when circuits are available within the newly entered cell.
LaMonte et al. (2004)				
Bergrath et al. (2012)	Portable data transmission unit (peeg-box) with four parallel data channels, video camera, audio communication devices	Mobile, real-time audio–video and still pictures	2G and 3G	Of the teleconsultations, 17% experienced technical failures related to partial dropouts of single applications and failed still picture and video transmission.
Liman et al. (2012)	Prototype mobile telemedicine device (VIMED CAR)	Mobile, real-time audio–video	3G	Of the teleconsultation cases reported, 60% could not be performed due to poor quality, loss, or absence of audio-video signal.
Van Hooff et al. (2013)	Commercially available hardware, web-based telemedicine platform	Mobile, real-time audio–video	4G	Of the cases, 12% experienced short freezing of the video (<5 s), 10% of cases noted suboptimal audio transmission and loss of signal in 1 session due to disconnection of the 4G USB dongle caused by vibration in the ambulance.
Eadie et al. (2015)	Tablet-based communications system (Omni-Hub), SIM card (Verizon), Motion X GPS, antennae	Mobile, real-time audio–video	2G and 3G	Of teleconsultations, 47% experienced occasional breaks or freezes but connection was re-established quickly and only minor delays. None of the teleconsultations were abandoned because of connectivity or other technical problems.
Wu et al. (2014)	Commercially available portable telemedicine unit (RP-Xpress), zoom camera and microphone with speakers, mobile hotspot (Verizon 4G LTE Jetpack)	Mobile, real-time audio–video	4G LTE	Of the teleconsultations, 15% experienced major technical complications; 7% were due to poor network connectivity, 5% were due to automatic software updates requiring rebooting and 3% involved an error in equipment charging.
Belt et al. (2016)	Commercially available portable telemedicine unit (RP-Xpress)	Mobile, real-time audio–video	4G	39% of teleconsults required reconnection; in all but two cases, connectivity was reestablished rapidly.
Chapman Smith et al. (2016)	Off the shelf, tablet-based telemedicine system (Cisco Jabber)	Mobile, real-time audio–video	4G	No major technical interruptions.
Valenzuela Espinoza et al. (2016)	Prototype platform using commercially available components including video camera (Mobotix), and IXSys software	Mobile, real-time audio–video	4G	Of teleconsultations, 6% experienced technical issues due to battery malfunction of the in-ambulance device.
Itrat et al. (2016)	Commercially available portable telemedicine unit (RP-Xpress), mobile CT scanner	Mobile, real-time audio–video	4G LTE	Of teleconsultations, 6% experienced technical difficulties. Most were due to pockets of poor network reception (5%), and a small portion were due to incompatibility of devices (1%). No video disconnection lasted longer than 60 s.
Lippman et al. (2016)	Off the shelf, tablet-based telemedicine system (Cisco Jabber)	Mobile, real-time audio–video	4G	Excluding one route (1/6) with poor transmission quality, 87.5% of test calls achieved bidirectional AV quality with paired ratings of 4 or higher Most common compliant associated with poor video quality was freezing of the image, with raters mentioning pixelation and loss of fluidity mentioned in two other poorly scored runs.
Barrett et al. (2017)	Off the shelf, tablet-based telemedicine system	Mobile, real-time audio–video	4G LTE	There were minor audio and video technical limitations noted in some cases (i.e., AV freezing), which occurred for a few seconds. Additionally, one teleconsultation noted that the video dropped twice during severe thunderstorms.
Wu et al. (2017)	Commercially available portable telemedicine unit (RP-Xpress and Maxlife)	Mobile, real-time audio–video	4G LTE	Of the teleconsultations, 2% experienced major technical complications. This includes a failure of the telemedicine camera as well poor internet connectivity.
Chapman Smith et al. (2019)	Commercially available tablet-based telemedicine system	Mobile, real-time audio–video	4G LTE	Of the teleconsultations, 9% were not completed—this was due to time constraints, persistent drops and difficulties initiating calls with one of the tablet devices.
Geisler et al. (2019)	Commercially available hardware (VIMED CAR), video camera (PTX), fixed microphone	Mobile, real-time audio–video	3G and 4G LTE	Of the cases, 7% were not completed due to technical difficulties (including failure of video connection).
Johansson et al. (2019)	Commercially available hardware	Mobile, real-time audio–video	2G, 3G, 4G & 4GLTE	Of the cases, 9% reported deficient (no communication possible) audio quality, as noted by the nurses and physicians.
Winter et al. (2019)	Commercially available hardware (VIMED CAR), video camera (PTX), fixed microphone	Mobile, real-time audio–video	3G and 4G	Connectivity was not available in 17% of 4G- and 15% of 3G- attempts with 6% simultaneous unavailability of both networks. Technical disturbances included fragmented/distorted AV transmission, delays in audio or video, noise interference and reduced video resolution
Al Kasab et al. (2021)	Commercially available hardware, web-based telemedicine platform	Mobile, real-time audio–video	##	No technical issues reported; however, authors did note, “remote neurological evaluations have inherent limitations and do not replace the need for in-person evaluation”

^*##*^Data not provided/data not available.

#### Clinical assessment

All of the studies but one reported some metrics regarding clinical outcomes and applicability. These studies involved a variety of different assessment tools as detailed in Table 4. Fourteen studies (74%) used some version of the NIH Stroke Scale. Two studies (11%) included a complete neurologic examination via the mobile telemedicine system. Two studies (11%) utilized a novel telestroke assessment called the Unassisted TeleStroke Scale (UTSS). The training level and role of the personnel that functioned as either telepresenters (i.e., an individual present with the patient who operate the telehealth equipment and assist the remote physician with the examination) or remote telestroke providers varied widely. An EMS provider served in the role of ambulance-based telepresenter in most of the studies (74%). One study using the UTSS (5%) required no assistance from a telepresenter. The majority of studies (89%) utilized a stroke physician or an advanced practice provider as the remote examiner. In four studies (21%), stroke physicians were supported by either a neuroradiologist or a technologist.

**Table 4:** Clinical features of reviewed studies.

**References**	**Mobile personnel**		**Remote personnel**	**Type of assessment**	**Mean time (min) to complete assessment**
	**Telepresenter**	**Telestroke recipient**			
LaMonte et al. (2000)	EMS Provider	Health Volunteers and Stroke Patients	Stroke Physicians	Restructured NIHSS	##
LaMonte et al. (2004)	EMS Provider	Stroke Nurse Practitioners	Stroke Physicians	Restructured NIHSS	17^†^
Bergrath et al. (2012)	EMS Physician	Acute Stroke Patients	EMS Physicians	Neurologic Examination and Stroke History Checklist	##
Liman et al. (2012)	Emergency Physician	Healthy Volunteers	Stroke Physicians	NIHSS	##
Van Hooff et al. (2013)	No Assistance from Third Party	Healthy Volunteers	UTSS-trained Examiners	UTSS	3.1^‡^
Eadie et al. (2015)	EMS Provider	Healthy Volunteers	Stroke Physician	Telestroke Checklist (includes NIHSS and mRS	11^†^
Wu et al. (2014)	EMS Provider	Stroke Nurses and Senior Neurology Residents	Stroke Physicians	Clinical Data Points and NIHSS	10^‡^
Belt et al. (2016)	EMS Provider	Acute Stroke Patients	Stroke Physicians	Neurological Evaluation	7.3^‡^
Chapman Smith et al. (2016)	EMS Provider (VA) and Stroke Physician (CA)	Medical Students and Healthy Volunteers	Stroke Physicians	NIHSS	##
Valenzuela Espinoza et al. (2016)	EMS Provider	Acute Stroke Patients	Stroke Physicians	GCS, UTSS, mRS and tPA Checklist	9^‡^
Itrat et al. (2016)	Registered Nurse or EMS Provider	Acute Stroke Patients	Stroke Physicians and Neuro-radiologists	NIHSS and Mobile CT scan	20^‡^
Barrett et al. (2017)	EMS Provider	Healthy Volunteers and Acute Stroke Patients	Telestroke Physicians	NIHSS	7.6^‡^
Wu et al. (2017)	EMS Provider or Nurse	Acute Stroke patients	Stroke Physicians	NIHSS	35.8^§^
Chapman Smith et al. (2019)	EMS Provider	Standardized Actors^¶^ and Healthy Volunteers	Stroke Physicians	NIHSS	9^‡^
Geisler et al. (2019)	Radiology Assistant	Patients with Neurological or Non-neurological Symptoms	Stroke Physicians	NIHSS	18.5^‡^
Johansson et al. (2019)	Prehospital Emergency Nurses	Acute Stroke Patients	Stroke Physicians	NIHSS	##
Winter et al. (2019)	EMS Provider	Standardized Actors^¶^	Stroke Physicians	Extended NIHSS	3G connection: 10^‡^ 4G connection: 9^‡^
Al Kasab et al. (2021)	EMS provider	Acute Stroke Patients	ED provider and Telestroke provider	NIHSS	##

NIHSS, National Institutes of Health Stroke Scale; UTSS, Unassisted TeleStroke Scale; mRS, Modified Rankin Scale; GCS, Glasgow Coma Scale; tPA, Tissue Plasminogen Activator.

^*##*^Data not provided/data not available.

^†^Minutes to the point of treatment.

^‡^Minutes to complete the assessment.

^§^Time from arrival of the MSU to the point of treatment.

^¶^Standardized actors are experienced patient actors, recruited through the medical school training program.

#### Simulations

Ten studies evaluated the use of a mobile telestroke platform during clinical simulations for a total of 306 encounters. One of the studies conducted clinical simulations within a mobile stroke unit and compared the technical parameters of 4G and 3G connectivity. Over half of the studies (70%) were able to perform >80% of their mobile teleconferencing encounters without prohibitive technical interruptions. There was variability in the evaluation of reliability of neurologic assessments, including agreement in the clinical diagnosis and treatment decisions as demonstrated in Table 5. Most of the studies (70%) reported the reliability of the mobile teleconferencing clinical assessment using either interrater reliability or intraclass correlation. Three studies revealed moderate to excellent reliability. Two studies evaluated the agreement in treatment decisions using different assessment techniques. Five of the studies conducted surveys with healthcare providers concerning satisfaction, AV quality, efficacy of providing valuable information, usability, and acceptability of technology with favorable responses.

**Table 5:** Reviewed studies involving clinical simulations.

**References**	**No. of subjects**	**Data network**	**Assessments completed**	**Comparison**	**Agreement**
LaMonte et al. (2000)	25	2G	Not provided	Not provided	Not provided
LaMonte et al. (2004)	12	2G	Not provided	Original NINDS training videotape through TeleBAT system vs. TV/VCR presentation	High interrater reliability
Liman et al. (2012)	30	3G	40%	Hospital-based stroke physician, emergency physician in the ambulance guide through telemedicine and “a posteriori” based on video documentation	Moderate to good interrater reliability
Van Hooff et al. (2013)	41	4G	96%	Remote physician vs. video documentation	Moderate to excellent interrater reliability
Eadie et al. (2015)	23	2G and 3G	100%	In motion vs. stationary	All cases correctly categorized according to potential for receiving tPA
Wu et al. (2014)	40	4G LTE	85%	Remote physician vs. recorded video and scripted scenarios	Moderate to excellent interrater reliability (ICC = 0.997). Matched real-time assessments for 88% of NIHSS (±2 points) and for 96% of clinical information
Chapman Smith et al. (2016)	27	4G LTE	100%	Remote vs. hospital bedside and ambulance bedside vs. remote	ICC = 0.96
Barrett et al. (2017)	3	4G LTE	100%	Feasibility of video call with remote physician	All 5 video calls were feasible
Chapman Smith et al. (2019)	65	4G LTE	91%	Remote physician vs. hospital bedside evaluation	Moderate interrater agreement (κ = 0.46)
Winter et al. (2019)	40	3G and 4G	3G: 85.0% • 4G: 92.5%	Video-conferencing quality of 3G vs. 4G via ambulance-based telestroke system on mobile stroke unit	3G validity: • NIHSS/eNIHSS sum score, ICC = 0.84 • eNIHSS reliability: ICC = 0.98 Reliability: • Thrombolytic treatment decision, κ = 1.0 • Relative contraindication, κ = 0.72 4G validity: • NIHSS/eNIHSS sum score, ICC = 0.89 • eNIHSS reliability, ICC = 0.99 Reliability: • Thrombolytic treatment decision, κ = 1.0 • Relative contraindication, κ = 0.60

NINDS, National Institute of Neurological Disorders and Stroke; “a posteriori”, a rating is by a stroke physician on the basis of video documentation; ICC, Intraclass correlation coefficient, a descriptive statistic that assesses agreement in the cases of two or more independent raters and measures the reliability of said raters; this statistic can range from 0 to 1 with 0 indicating no reliability among raters and 1 indicating perfect reliability among raters; eNIHSS, Expanded National Institutes of Health Stroke Scale; κ, Cohen's kappa coefficient, a statistic utilized to measure reliability among raters for qualitative items; a κ <0.40 represents “poor” agreement.

#### Live patient testing

Seven studies evaluated the use of a mobile telestroke platform in a prospective feasibility study with actual patients for a total of 194 encounters. All of the studies except one reported the percentage of mobile teleconferencing encounters without prohibitive technical interruptions with all noting >80% of their encounters completed. There were variations in the measured outcomes of each of the studies noted in Table 6. Results mainly focused on the clinical value of conducting an ambulance-based telestroke assessment, feasibility of assessment, and accuracy of the prehospital diagnosis. Two studies demonstrated a reduction in door-to-needle times when compared with standard bedside assessments.

**Table 6:** Reviewed studies involving live clinical testing on patients.

**References**	**No. of subjects**	**Data network**	**Assessments completed**	**Comparison**	**Results**
LaMonte et al. (2000)	6	2G	Not provided	Not provided	Not provided
Bergrath et al. (2012)	12	2G and 3G	83%	Tele medically assisted prehospital care vs. local standard EMS care	•Subjective clinical value reported as helpful to very helpful by 85% of participants. •No significant differences in time measurements. •Prehospital stroke diagnosis confirmed: telemedicine group, 61%; control group, 67%.
Belt et al. (2016)	89	4G	100%	With vs. without in-transit telestroke	•“Door-to-needle” time: telestroke, 28 min; control, 34 min. •Negative MRI: telestroke, 20%; control, 23%.
Valenzuela Espinoza et al. (2016)	16	4G	96%	Remote vs. in-hospital diagnosis	•Prehospital stroke diagnosis concordant with inpatient diagnosis in 83% of cases. •No stroke diagnosis was missed during in-ambulance telestroke consultations.
Barrett et al. (2017)	11	4G LTE	91%	Primary endpoint: NIHSS assessment of stroke patient en route to facility	•NIHSS obtained in 91% of cases.
Johansson et al. (2019)	11	2G, 3G, 4G, and 4GLTE	100%	Primary endpoint: assessment of complementary statements of prehospital emergency care nurses based on quantitative and qualitative methods	•Video system worked correctly in all cases. •Physicians considered that the imaging quality in the assessment procedure was more than satisfactory (100% very good and good), without the need for multiple assessment attempts. •In only 1 case was the audio considered deficient.
Al Kasab et al. (2021)	49	##	100%	Time metric between patients evaluated via telestroke consults in the emergency medical services unit and received tPA vs. 52 patients who received tPA via standard	•Among the 44 patients transferred to the NSC, 15 (34.1%) received tPA on arrival. During the same period, 52 patients received tPA through STS.
				telestroke consults (STS) performed in the same nearest stroke center (NSC)	consults at the same NSC. Compared to STS patients, TEMS patients had shorter door-to-needle (DTN) times (21 vs. 38 min, *P* < 0.001).

^*##*^Data not provided/data not available.

#### Mobile stroke units with live patients

Three studies have evaluated the use of mobile ambulance-based telestroke in mobile stroke units prospectively (Table 7). There were a total of 377 encounters with all studies reporting a successful assessment completion rate of at least 85%. Two of the studies compared the remote telemedicine evaluation with an on-board bedside evaluation. One compared remote telemedicine evaluation with the emergency room bedside evaluation. Two of the studies evaluated the reliability of the neurological assessment with a high interrater reliability and an NIHSS intraclass correlation of at least 0.87. One study noted a reduction in onset-to-needle time of 26 min compared to control.

**Table 7:** Reviewed articles involving live prehospital telestroke on mobile stroke unit (MSU).

**References**	**No. of subjects**	**Data network**	**Assessments completed**	**Comparison**	**Results**
Itrat et al. (2016)	100	4G	99%	Patients evaluated and treated on MSU vs. patients brought via ambulance to ED	•16% of patients received tPA on MSU. •MSU reduced time to tPA by 26 min (MSU, 32 min; control, 58 min).
Wu et al. (2017)	174	4G LTE	98%	Telemedicine vs. onboard evaluation	•Decision regarding tPA agreed in 88% of cases. •Technical problems prevented decision from telemedicine vascular neurologist in 2% of cases. •NIHSS intraclass correlation, 0.88.
Geisler et al. (2019)	103	3G and 4G LTE	87%	Telemedicine vs. onboard evaluation	•High interrater reliability. •NIHSS intraclass correlation, 0.87. •A decision for IV thrombolyis was made for 18 patients by the MSU neurologist, while the remote neurologist recommended IV thrombolysis in 16 of these patients (*k* = 0.93).

## Discussion

This literature review suggests that ambulance-based telestroke is a feasible and reliable method for prehospital stroke assessment. Since the early 2000's, the technical feasibility of conducting real-time, mobile telemedicine in a moving ambulance has largely improved due to refinements in mobile technology and the wider availability of high-speed mobile broadband. Nevertheless, advances in acute stroke treatments have added complexity to stroke systems of care and incurred an even greater requirement on the accuracy of prehospital stroke diagnosis to inform triage decisions, particularly for patients with large vessel occlusions. However, there remains a great deal of variability in the practice and usability of ambulance-based telestroke given the challenges of integrating in modern emergency stroke workflows.

Here we described the current literature regarding the accuracy and reliability of ambulance-based telestroke technologies and clinical assessments. Despite the evidence supporting the feasibility and reliability, the medical literature is still lacking in several areas regarding the use of such novel technology in the prehospital arena. One area is related to the significant amount of variability within the studies evaluated. There were variations in the type of equipment, stroke assessment, clinical evaluator, methodology, and outcomes assessed. While the heterogeneity among studies makes comparisons challenging, this variability reflects the challenges of real-world prehospital clinical practice.

Despite these significant variations among the studies in this review, the feasibility, reliability, and validity data remain favorable as technology has improved. In a recent cluster randomized controlled trial comparing the diagnostic accuracy of prehospital telestroke with that of in-person emergency department (ED) assessment, telestroke was 100% accurate in predicting reperfusion candidates compared with preimaging ED examination. Additionally, telestroke was found to be 80 and 88.6% accurate in predicting intervention with IV thrombolytic and endovascular treatment, respectively, suggesting that telestroke can be an effective option to reduce delays to definitive stroke care (Scott et al., 2022).

In addition, more than half of the studies reviewed were conducted within a simulated environment. These studies opted for this style of investigation for several reasons. The condition of real patients can change during transport, making it impossible to define a definitive stroke scale score with which to compare telestroke assessments. Simulation offered these investigators the standardization needed to draw conclusions about the technology and assessments used in these studies. However, the use of standardized patients creates gaps in knowledge of the performance of a telestroke system. For example, the simulated scenarios tested may not have captured the variety of patient presentations that stroke providers encounter in clinical practice. Also, the use of standardized patients may have facilitated an easier exam for the providers. Some studies used medical students, nurses, or neurology residents as actors. A standardized patient that has a sophisticated understanding of stroke presentations might bias toward easier assessments.

Furthermore, an important factor that may impact the feasibility, reliability, and accuracy of stroke assessments is transport setting (i.e., interfacility and prehospital). Specifically, telestroke in a prehospital setting may have a higher rate of stroke mimics compared to those undergoing interhospital transfer, where patients often have screened positive for stroke and undergo transport to a comprehensive stroke center. Notably, in our review, the transport setting in a majority of studies was pre-hospital, where the need for telestroke as a triage tool is likely greater. However, telestroke use in interfacility transfer can be useful to expedite in -hospital workflows to reduce further delays to treatment.

Regarding technical feasibility of ambulance-based telemedicine, the majority of the barriers encountered were related to modifiable human and operational errors (e.g., unscheduled software updates, unchanged batteries/devices, and attempted use of incompatible equipment). Examples included a case in which an automatic software update required rebooting of the device, while another case was infeasible due to the Wi-Fi hotspot being out of battery after being charged with an improper charger. Although these instances prevented the completion of the prehospital assessment, they represent modifiable factors that can be addressed easily with a more standardized and streamlined approach to integration and prehospital workflow.

Suffice it to say, human factors and ease-of-use play a significant role in the sustainability of telemedicine systems in the healthcare environment, particularly in time sensitive scenarios such as acute stroke (Rogers et al., 2021). Healthcare systems seeking to implement prehospital telemedicine may need to rely on technical support from established telemedicine programs to effectively process and interpret mobile telestroke assessments in real time. Alternatively, as used in the emergency room setting during times of technical failure, mobile phone devices could be used in cases of telemedicine failure. However, further investigation is required to understand best practices for these mobile devices in this setting.

An in depth understanding of wireless network capabilities within a particular catchment region is also crucial to the success of ambulance-based telestroke services. While the potential for real time ambulance-based telemedicine has been made possible by the advancement of cellular networks, mobile broadband remains fragmented in many parts of the world. Clearly, the sustainability of ambulance-based telemedicine programs will require partnering with local telecommunications providers and government officials to strengthen regional broadband capability, particularly for rural and underserved areas.

Integrating ambulance-based telestroke capability for healthcare systems will require additional cost-utility analysis. Only a few studies commented on the cost of outfitting an ambulance with a telemedicine system, ranging widely depending on the type of technology and human resources employed within each system. Hopefully, the ongoing advancement of mobile health technology and access will continue to provide cheaper options for performing ambulance telemedicine encounters, including the use of commercially available platforms incorporating mobile phones and tablet-based devices. Ambulance-based telemedicine has also enhanced the cost-effectiveness of implementing CT-capable mobile stroke units as well, becoming an essential component of the prehospital clinical assessment needed to avoid overextending costly-human resources on these units.

We acknowledgethat our study has several limitations. Individual patient data and subgroup analysis were not possible, as a study-level analysis was performed. Furthermore, as mentioned, the included studies were heterogenous in type of equipment, stroke assessment, clinical evaluator, methodology, and outcomes assessed, making full interpretation of the available data challenging. Additionally, since the time of search for this review, additional studies have evaluated the feasibility and reliability of ambulance-based telestroke that were not included in this review. However, overall, these studies do endorse the feasibility and reliability of telestroke, finding that telestroke does allow for correct stroke and large vessel occlusion identification in the majority of cases (https://pubmed.ncbi.nlm.nih.gov/37941577/, https://pubmed.ncbi.nlm.nih.gov/36240100/, https://pubmed.ncbi.nlm.nih.gov/35720454/, https://pubmed.ncbi.nlm.nih.gov/33690029/). A majority of these more recent studies also focus on reviewing telestroke in the context of improving times to reperfusion in the setting of acute stroke interventions (i.e., IV thrombolytics and endovascular treatment).

## Conclusion

This topical review using a systematic search strategy suggests that ambulance-based telestroke is a feasible strategy to provide reliable and accurate stroke assessment to patients in the prehospital and interfacility settings. Further prospective research is needed to further assess cost-effectiveness and utility for prehospital stroke triage, particularly for endovascular therapy, and confirm generalizability of implementation across diverse geographic regions and prehospital systems of care.

## Data availability statement

The original contributions presented in the study are included in the article/supplementary material, further inquiries can be directed to the corresponding author.

## Author contributions

SC: Conceptualization, Data curation, Formal analysis, Methodology, Project administration, Supervision, Writing—original draft, Writing—review & editing. TM: Data curation, Writing—original draft, Writing—review & editing. ND: Data curation, Writing—original draft, Writing—review & editing. JN: Data curation, Formal analysis, Writing—original draft, Writing—review & editing. NR: Data curation, Writing—original draft, Writing—review & editing. HP: Data curation, Writing—original draft, Writing—review & editing. MD: Data curation, Writing—original draft, Writing—review & editing. AS: Conceptualization, Supervision, Writing—original draft, Writing—review & editing.

## Appendix

### Definitions

The authors of this review agreed upon the following definitions to be used in the reporting of the data found in the included articles. These definitions were used to categorize studies and analyze data.

**Ambulance-based telestroke:** any system designed to allow remote personnel with stroke expertise to examine a patient in an ambulance using audio, video, photo, or other means of digital information transfer.

**Ambulance-based telestroke technical feasibility study:** A study that investigates the likelihood that the technological solutions employed by an experimental ambulance-based telestroke system could support use during real-world emergencies.

**Ambulance-based telestroke simulation study:** A study that makes use of actors to play the role of stroke patients to investigate the feasibility of an ambulance-based telestroke system.

**Ambulance-based telestroke assessment reliability/validity study:** A study that investigates the results of telestroke assessment by measuring agreement between raters (reliability) or agreement with a gold-standard method of stroke assessment

**Ambulance-based telestroke observational study:** A study that employs telestroke in an emergency medical system and gathers data from telestroke encounters.

**Mobile stroke unit:** an ambulance that furnishes services to diagnose, evaluate, and/or treat symptoms of an acute stroke (CITE).

**Stroke Physician:** expertise in stroke.

**EMS provider:** An individual trained specifically in the prehospital treatment and transport of patients, level of credentialing and expertise is variable.

**Prehospital ambulance:** a vehicle that responds to emergency calls in the community and transports patients to the hospital prior to tPA treatment decisions being made.

**MSU:** ambulances that respond to emergency calls however they are designed to obtain CT images and deliver IV thrombolysis in the field.

**Interfacility ambulance:** An ambulances that does not respond to emergency calls in the community but rather transports patients from one healthcare facility to another.
